# Parazentrale Skotome bei COVID-19-Infektion

**DOI:** 10.1007/s00347-022-01726-z

**Published:** 2022-09-09

**Authors:** Bogdana Kovalchuk, Lucy J. Kessler, Gerd U. Auffarth, Christian S. Mayer

**Affiliations:** 1 Univ.-Augenklinik, Universitätsklinikum Heidelberg, Im Neuenheimer Feld 400, 69120 Heidelberg, Deutschland; 2 Augenklinik am Ortenau Klinikum Offenburg-Kehl, Weingartenstr. 70, 77654 Offenburg, Deutschland

## Anamnese

**Figure 1:**
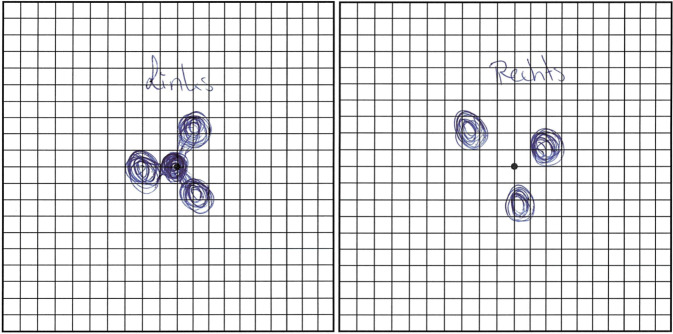

Eine 16-Jährige sonst gesunde Schülerin stellte sich im Februar 2022 in unserer Notfallambulanz vor. Sie berichtete über die Wahrnehmung von parazentralen Skotomen an beiden Augen („Flecken wie Mickey-Maus-Ohren um das zentral noch scharfe Sehen“, vgl. Abb. 1), welche 10 Tagen zuvor zum ersten Mal aufgetreten seien und sich seither leicht in ihrer Form verändert hätten. Sie sei in der vergangenen Woche an COVID-19 mit mildem Verlauf erkrankt gewesen, die Symptome an den Augen seien ungefähr zeitgleich mit den Atemwegssymptomen aufgetreten. Kopfschmerzen oder anderweitige Beschwerden bestünden nicht, die Skotome seien jedoch störend.

## Klinischer Befund

**Figure 2:**
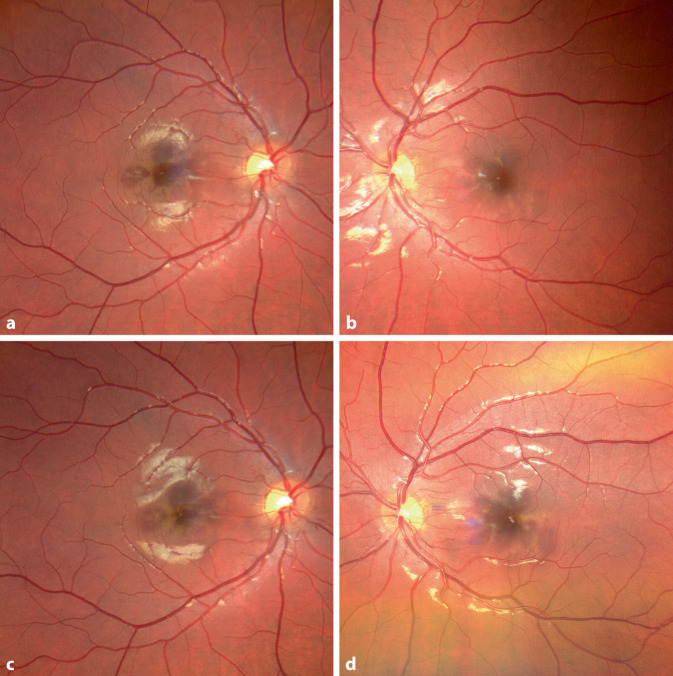

Bei Erstvorstellung zeigte sich ein bestkorrigierter Visus von rechts 0,8 und links 0,63 dezimal. Die Vorderabschnitte stellten sich regelrecht dar, fundoskopisch zeigten sich sehr dezente, kaum sichtbare, grau-bräunliche petaloide perifoveale Läsionen (vgl. Abb. 2a, b) bei ansonsten regelrechtem Fundusbefund.

## Diagnostik

**Figure 3:**
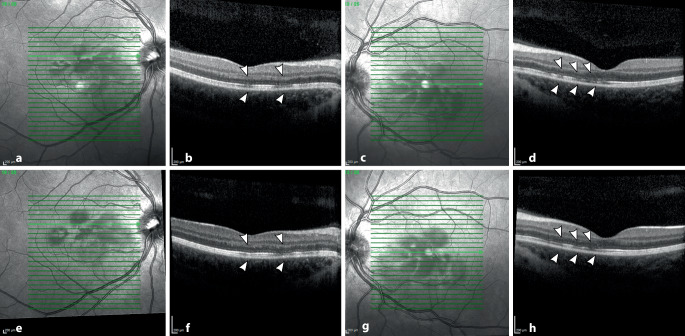

**Figure 4:**
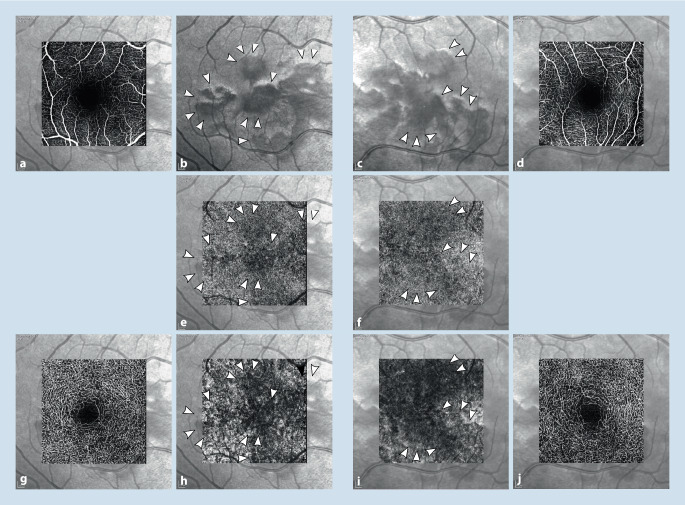

In der optischen Kohärenztomographie („spectral-domain optical coherence tomography“ [SD-OCT]) stellten sich in der Makula beidseits Unterbrechungen der ellipsoiden Zone (EZ) dar (Abb. 3b, d). Im Infrarotbild zeigten sich diese als petaloide perifoveale Läsionen, welche mit den bräunlichen Läsionen in der Fundoskopie korrelierten (Abb. 3a, c). In der Autofluoreszenz waren keine Auffälligkeiten festzustellen, ebenso nicht in den oberflächlichen Schichten der OCT-Angiographie (OCTA) (vgl. Abb. 4a, d, g, j). In Choriokapillaris und Choroidea hingegen ließen sich verminderte Flusssignale in Korrespondenz mit den petaloiden Defekten nachweisen (Abb. 4e, f, h, i). Bei für die Erkrankung bereits pathognomonischen Befunden wurde auf die Durchführung einer Fluoreszeinangiographie verzichtet.

## Wie lautet Ihre Diagnose?

## Therapie und Verlauf

In Zusammenschau aller Befunde stellten wir die Diagnose einer akuten makulären Neuroretinopathie (AMN). Nach einer Woche berichtete die Patientin über eine Formveränderung der Skotome bei unveränderten Visuswerten und unveränderten Untersuchungsbefunden. Nach einem Monat wurde eine leichte Besserung im Sinne einer Abblassung der Skotome berichtet mit einem bestkorrigierten Visus von 0,8 beidseits. Die Integrität der ellipsoiden Zone zeigte in der OCT eine partielle Restitution.

## Diskussion

Bei der akuten makulären Neuropathie handelt es sich um ein insgesamt seltenes Krankheitsbild (Prävalenz < 1:100.000 [10]), welches vorwiegend junge kaukasische Frauen betrifft und meist im Zusammenhang mit einer grippeartigen Infektion, der Einnahme von Ovulationshemmern oder vasokonstriktiven Substanzen (Epinephrin, Ephedrin, Amphetamine …) beschrieben wird [2, 3, 5]. Die genaue Ätiologie ist bisher nicht bekannt, ausgegangen wird jedoch von einem mikrovaskulären ischämischen Ereignis in den tiefen retinalen Schichten, welches zu hyperreflektiven Banden in der äußeren plexiformen („outer plexiform layer“ [OPL]) und äußeren nukleären („outer nuclear layer“ [ONL]) Schicht und Disruption der ellipsoiden Zone in typischen petaloiden (blüten-, keil- oder tropfenförmigen) um die Makula angeordneten Läsionen führt [2, 10]. Im Rahmen von COVID-19 werden eine inflammationsbedingte Hyperkoagulabilität und Thrombogenität oder eine durch ACE-2-Inaktivierung bedingte Störung des Renin-Angiotensin-Systems (RAS) und damit Vasokonstriktion diskutiert [11].

OCT und Infrarotdarstellung sind für die Diagnose wegweisend, da die dezenten Läsionen fundoskopisch leicht zu übersehen sind. Neuere Studien zeigen verminderte Flusssignale in der OCTA, zumeist im tiefen retinalen Gefäßplexus [3, 6, 7, 9] und/oder der Choriokapillaris [5] und inneren Choroidea [9]. In unserem Fall zeigte sich ein verminderter Blutfluss in Choriokapillaris und Choroidea.

**Diagnose:** Akute makuläre Neuroretinopathie (AMN) im Zusammenhang mit einer COVID-19-Infektion

Im Rahmen der COVID-19-Pandemie kann die AMN im Zusammenhang mit einer Infektion oder einer Impfung auftreten und den Atemwegssymptomen gelegentlich auch vorausgehen [10]. In den allermeisten bisher beschriebenen Fallberichten besteht mindestens ein weiterer Risikofaktor, meist eine orale Kontrazeption [3, 5]. Da diese in dem hier vorliegenden Fall verneint wurde und keine weiteren Komorbiditäten bekannt waren, ist davon auszugehen, dass eine AMN auch durch COVID-19 allein ausgelöst werden kann.

Die Literaturrecherche zeigt zudem, dass im Rahmen von COVID-19 eine AMN gehäuft auch bei älteren Personen zu beobachten ist [4, 10]. Bei Sehstörungen im Rahmen von COVID-19 sollte bei älteren Patienten daher nicht nur an die Conjunctivitis sicca, welche die häufigste Beteiligung von COVID-19 am Auge darstellt [3], sondern auch an eine mögliche AMN gedacht werden. Die Symptomabfrage mit dem Amsler-Gitter (der Patient wird gebeten, die wahrgenommenen Skotome auf einem Amsler-Gitter einzuzeichnen) kann bei Immobilität oder unter Isolationsbedingungen hilfreich sein, wenn keine OCT zur Verfügung steht.

Eine spezifische Therapie für die AMN gibt es nach heutigem Kenntnisstand nicht. Ob die Reduktion der Risikofaktoren, also beispielsweise das Pausieren der oralen Ovulationshemmung oder vasokonstriktiven Medikation sowie Meiden von Koffeinkonsum, einen Einfluss auf den Krankheitsverlauf hat, ist bisher nicht bekannt [10]. In Einzelfällen wurde eine Verbesserung der Durchblutung der inneren Netzhautschichten unter oraler Prednisolon-Therapie postuliert [5, 8, 9], allerdings liegen in diesen Berichten keine Kontrollen zum Vergleich vor. Wie in dem hier beschriebenen Fall kommt es in der Regel zu einer Spontanremission innerhalb einiger Monate [1]. Regelmäßige augenärztliche Kontrollen inklusive der OCT dienen dem Ausschluss anderer retinaler COVID-Manifestationen, wie z. B. retinaler Blutungen oder Gefäßverschlüsse [3].

## Fazit für die Praxis

- Die akute makuläre Neuroretinopathie (AMN) kann auch im Zusammenhang mit einer COVID-19-Infektion oder -Impfung auftreten und äußert sich durch die meist beidseitige Wahrnehmung von parazentralen relativen Skotomen.
- Pathognomonisch sind bräunliche petaloide perimakuläre Läsionen am Fundus, welche sich am besten in der Infrarotbildgebung darstellen und mit Diskontinuitäten der ellipsoiden Zone sowie ggf. Hyperreflektivitäten der ONL („outer nuclear layer“) und OPL („outer plexiform layer“) in der OCT („optical coherence tomography“) korrelieren.
- Eine spezifische Therapie existiert aktuell nicht, in der Regel kommt es zu einer Spontanremission innerhalb einiger Monate.
- Auch ältere Patienten können im Rahmen von COVID-19 an einer AMN erkranken. Steht keine OCT-Diagnostik zu Verfügung, so kann die Symptomabfrage mittels Amsler-Gitter hilfreich sein.
